# Comparison of 4D and 2D phase contrast magnetic resonance imaging of the great mediastinal vessels

**DOI:** 10.1186/1532-429X-15-S1-P35

**Published:** 2013-01-30

**Authors:** Uta Preim, Franziska Hause, Lukas Lehmkuhl, Bernhard Preim, Andreas Greiser, Matthias Grothoff, Matthias Gutberlet

**Affiliations:** 1 Radiology, University of Leipzig, Heart Center, Leipzig, Germany; 2 Faculty of Computer Sciences, University of Magdeburg, Magdeburg, Germany; 3 Siemens AG Medical Solutions, Siemens AG, Erlangen, Germany

## Background

Two-dimensional (2D) phase contrast (PC) magnetic resonance imaging (MRI) enables non-invasive measurements of forward and backward flow, shunt volumina and peak velocity. It is an important tool in the diagnosis and follow-up of patients with congenital or acquired cardiovascular diseases. However, planning and repeated acquisition of multiple 2D measurements is time-consuming and data analysis is restricted to those areas that were targeted during the scan. Four-dimensional (4D) PC MRI enables flow assessment through all cardiac valves and mediastinal vessels during one acquisition. The purpose of our study was to compare the 4 dimensional (4D) phase contrast (PC) technique against the established 2 dimensional (2D) technique and test the feasibility of the 4D technique.

## Methods

We included 3 volunteers and 10 patients. 2D PC measurements were performed either at a 1.5T or a 3.0T and 4D flow measurements at a 3.0T MR device. Statistical analysis included the Wilcoxon test, Pearson correlation coefficient, linear regression analyses and Bland-Altman plots.

## Results

Flow volumes were obtained with 2D and 4D measurements in 25 positions. They showed good agreement without significant differences (p=0.16). There was a strong correlation between 2D and 4D flow volumes (r=0.9). The correlation coefficient for forward flow was 0.6 and for backward flow 1.0.

Peak velocity values were obtained with 2D and 4D measurements in 21 locations. 2D and 4D measurements showed no significant differences (p=0.054) and a good correlation (r=0.8). The Bland-Altman test showed good agreement for flow volumes and peak velocity. Ratios of pulmonary to aortic stroke volumes for 2D and 4D flow measurements in three volunteers showed consistent values.

## Conclusions

The 4D flow technique is suitable for clinical use and shows reliable results compared with the gold standard 2D measurement.

## Funding

There was no funding.

